# Effects of simulated warming and litter removal on structure and function of semi-humid alpine grassland in the Qinghai-Tibet Plateau

**DOI:** 10.3389/fpls.2025.1567414

**Published:** 2025-05-15

**Authors:** Guomin Xue, Lihua Tian, Jingxue Zhao

**Affiliations:** ^1^ Sichuan Zoige Alpine Wetland Ecosystem National Observation and Research Station, and College of Grassland Resources, Southwest Minzu University, Chengdu, China; ^2^ State Key Laboratory of Herbage Improvement and Grassland Agro-Ecosystems, and College of Ecology, Lanzhou University, Lanzhou, China

**Keywords:** alpine grasslands, experimental warming, ecosystem multifunctionality, litter removal, plant diversity

## Abstract

Climate warming and human activities are modifying plant litter inputs in alpine grasslands, which is predicted to affect ecosystem structure and function. However, the effects of plant litter removal and warming as well as the combined impacts on the ecological functions of alpine grasslands are not well understood. A field experiment was conducted to investigate the effects of experimental warming, litter removal, and their interaction on ecosystem multifunctionality (EMF) of alpine grasslands. Our results demonstrated a significant decrease in plant diversity (*p* < 0.05) and vegetation cover (*p* < 0.01) under experimental warming treatment, whereas the richness index (*R*) and belowground biomass (BGB) significantly increased under litter removal treatment (*p* < 0.05). The interaction effect of experimental warming and litter removal results in a neutralizing effect on the ecological functions in alpine grasslands. Meanwhile, the EMF tended to increase under all treatments of experimental warming, litter removal, and experimental warming-litter removal. However, there are differences in the response of aboveground and belowground multifunctionality to experimental warming and litter removal. The aboveground ecosystem multifunctionality (AEMF) showed a decreasing trend, while belowground ecosystem multifunctionality (BEMF) increased significantly (*p* < 0.01) under the experimental warming treatment. In contrast, AEMF and BEMF showed an increasing trend in litter removal treatment. In addition, the study found that litter removal could alleviate the negative effect of experimental warming on multiple ecological functions. These research findings can serve as a reference for maintaining ecosystem functions in alpine grasslands under climate change conditions and provide effective measures to enhance the capacity of grassland ecosystems to respond to climate change. The application of appropriate litter management measures and other nature-based solutions (NbS) to improve ecosystem functions, aiming to adopt sustainable approaches to address environmental challenges, holds significant importance for ecological conservation.

## Highlights

- Litter removal could alleviate the negative effect of experimental warming on aboveground ecosystem multifunctionality.
- There are differences in the response of aboveground and belowground multifunctionality to experimental warming and litter removal.
- Changes in ecosystem functioning due to experimental warming and litter removal are primarily driven by plant diversity, productivity, and soil nutrients.

## 1 Introduction

The interference of climate change affects ecosystem functions, specifically reflected in the relationship between climate-determined ecosystem functions and their driving factors (Jing et al., 2015). Similarly, global temperature changes, drought severity, and historical climate are important driving factors for ecosystem functioning (Ye et al., 2019). In the background of temperature changes, community structure and composition serve as the foundation of ecosystem functions, specifically reflected in changes in vegetation species and functional diversity, which may affect the potential ecosystem feedback to climate change (Ganjurjav et al., 2018; Shangguan et al., 2024). The relevant research indicated that warming affects plant growth and development by altering soil water and temperature, which in turn changes interspecies relationships within communities (Niu and Wan, 2008), consequently affecting the structure and composition of plant communities (Young et al., 2024). Among others, warming can affect soil moisture conditions and further contribute to the reduction of ecosystem functions through a decrease in plant diversity and vegetation productivity (Ma et al., 2023). Studies also indicated that the changes in temperature affect ecosystem functioning by altering the composition of grassland plant communities (Zhu et al., 2024). More specifically, warming affects grassland primary productivity by influencing plant community composition (Zhu et al., 2016), and this further affects the ecosystem functioning.

The global grasslands are not only experiencing severe climate change, but also withstanding high-intensity grazing activities (Fekete et al., 2016). Grazing is the main utilization method for grassland ecosystems, and long-term overgrazing will significantly compromise the functioning and structure of grassland ecosystems by reducing biodiversity, litter inputs, and plant productivity (Liu et al., 2023a). Meanwhile, the accumulation of litter was always considered a limiting factor for plant symbiotic structures and an important link between productivity, community, and ecosystem processes. A previous study suggested that overgrazing impacts on grassland ecosystem functions are mainly influenced by the reduction of litter inputs (Weltzin et al., 2005). Specifically, the indirect impact of grazing on plant communities is achieved by the litter removal, which leads to changes in species composition (Wang et al., 2011). Human disturbances such as burning and grazing combined with climate change factors like drought can significantly reduce plant litter production, thereby modifying plant diversity and subsequently affecting productivity and ecosystem functions (Zhang et al., 2023a; Liu et al., 2023b). Changes in plant litter can also impact ecosystem function by regulating soil temperature, moisture, and nutrient availability (Liu et al., 2023c). Specifically, litter removal can affect ecosystem functions through reductions in soil temperature and alterations in soil carbon and nitrogen content (Yan et al., 2018; Osborne et al., 2021). Sustainable management and nature restoration measures such as moderate grazing and litter removal can be incorporated into Nature-based Solutions (NbS). Studies have suggested that such restoration measures can enhance ecosystems by reducing external disturbances while leveraging ecosystem resilience and natural succession (Hua et al., 2022). However, the impact of reduced plant litter on ecosystem function is easily confused with the impacts of other disturbances (Chen et al., 2024). Therefore, it is crucial to understand how the loss of plant litter affects the response of grasslands to warming.

The Qinghai-Tibetan Plateau (QTP) is a key ecological region in China, with alpine grasslands representing its most important ecosystem. In recent years, the alpine grasslands on the QTP have been experiencing more rapid climate warming and higher-intensity livestock grazing than the global average rates (Yang et al., 2014), leading to a substantial impact on ecosystem functions. The alpine grassland ecosystem on the QTP is becoming increasingly vulnerable (Wang et al., 2024). Studies have shown that experimental warming significantly impacts the biological processes, structures, and functions in alpine grasslands (Liu et al., 2018; Zhao et al., 2019, 2024). Meanwhile, ecological functions are negatively responded to the accumulation of plant litter (Zhang et al., 2022). The accumulation of litter in grassland ecosystems influences community productivity, diversity, and species composition by affecting soil moisture and temperature (Hou et al., 2019), further impacting the ecosystem functions of alpine grasslands. Early research primarily focused on single ecosystem functions, such as productivity, litter decomposition, soil nutrients, and biodiversity. However, real-world ecosystems can provide multiple ecosystem functions simultaneously, with complex interactions such as promotion or antagonism occurring between these ecosystem functions (Byrnes et al., 2014). Therefore, ecologists have proposed the concept of ecosystem multifunctionality (EMF) in recent years, and research on the relationship between biodiversity and EMF has gradually increased (Van Der Plas, 2019; Duffy et al., 2017). Nevertheless, most research on multiple ecosystem functions has focused on overall EMF. The effects of warming, litter removal treatments, and their interactions on the functioning of the aboveground and belowground ecosystems remain largely unknown.

In this study, we hypothesize that experimental warming will decrease ecosystem functions, while litter removal will mitigate these effects by improving plant diversity, productivity, and soil conditions. Field experiments were conducted to investigate the effects of experimental warming and litter removal on the ecosystem structure and function of alpine grassland using four different treatments: control, experimental warming, litter removal, and experimental warming-litter removal. The main purpose of this study is to determine the changes in the aboveground and belowground ecological functions of alpine grasslands caused by experimental warming and litter removal, as well as their interaction, and to explore the key factors affecting the dynamic changes in ecological functions. This study is of great significance for understanding the impacts of global climate change and human activities on the ecological function of alpine grasslands, while providing measures and a theoretical basis for adopting natural restoration measures to improve ecosystems.

## 2 Materials and methods

### 2.1 Site description

**Figure 1:**
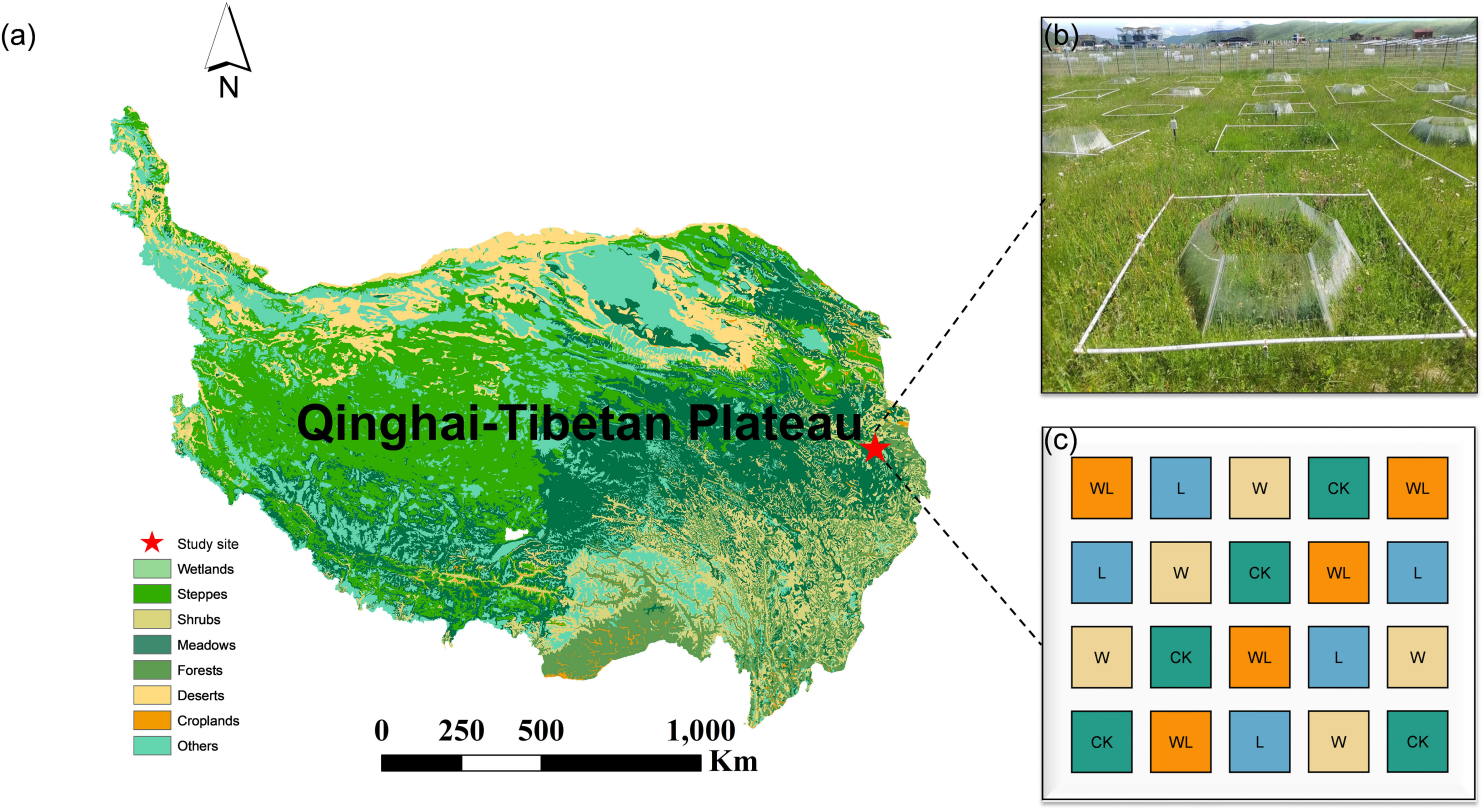
**(a)** Location of study area in Hongyuan county on the eastern Qinghai-Tibetan Plateau. **(b)** The landscape of the present litter removal, experimental warming and experimental warming-litter removal treatments experiment. **(c)** The experimental design of the litter removal, experimental warming and experimental warming-litter removal treatments.

The study was conducted at the Sichuan Zoige Alpine Wetland Ecosystem National Observation and Research Station, located in Hongyuan County, Sichuan Province, on the eastern edge of the QTP (**Figure 1**). The average altitude of the region is 3,507 m, and the average annual temperature is 1.5°C. January is the coldest month, with an average temperature of -9.5°C, while July is the hottest month, averaging 11°C. The annual rainfall reaches 749 mm, and 80% of the rain falls between May and September. The dominant vegetation species including *Anemone rivularis, Elymus nutans*, *Agrostis gigantea*, *Carex setschwanensis*, *Carex parvula*, *Oxytropis kansuensis*, and *Koeleria cristata.* The main soil type is alpine meadow soil, with a high organic matter content and a weakly acidic pH.

### 2.2 Experimental design

In November 2018, we conducted a rigorous field survey to select a typical homogeneous area representing the main characteristics of alpine meadows. Subsequently, we established a randomized complete block design experiment with experimental warming and litter removal treatments, and annual monitoring was conducted from 2019 to 2023. In this experiment, open-top chambers (OTCs) were used to simulate climate warming. The OTCs are constructed from polycarbonate panels (transmittance >95%), which enhance the penetration of solar radiation through the transparent panels while blocking wind to minimize heat loss, thereby achieving a warming effect, and the average surface temperature within the warming range of the OTCs increased by 1.31 (± 0.3) °C compared to the control. The experiment has 4 treatments (CK, W, L, WL, representing control, experimental warming, litter removal, and experimental warming-litter removal, respectively) and 5 replicates. The experimental area was divided into 20 quadrats (4 treatments × 5 replicates), each with an area of 4 m^2^ (2 m × 2 m). The litter removal treatment was carried out annually during the withering period, with both upright and fallen plant litter removed completely in each litter removal plot and the experimental warming-litter removal plot.

### 2.3 Vegetation and soil sampling

A field vegetation community survey and soil sampling were conducted during the peak period of the growing season from 2021 to 2023. Individual plants were identified at the species level in each of the quadrats. The coverage and natural height of each species were measured for each species within the sample quadrat. After the vegetation community survey, aboveground plant tissue was harvested from the sample quadrat to obtain the aboveground biomass (AGB). The soil samples were collected using a soil drill and sieved through a 2 mm mesh, and the root systems were subsequently washed with flowing water for belowground biomass (BGB) calculation. The AGB and BGB samples were weighed after oven-drying for 48 h at 65°C.

Bulk density was taken by a bulk density drill, and the soil moisture content (SM) was oven-dried to a constant weight treatment at 105°C for 72 hours. Soil samples of 0–10 cm depths were collected from the four corners of the quadrats for measurement of soil inorganic nitrogen (NH_4_^+^−N; NO_3_^−^−N), soil pH (pH), soil total nitrogen (STN), soil organic matter (SOM). The soil pH was measured with a pH meter. The SOC and STN contents were measured using the elementary analyzer (Vario EL III, Elementar, Germany). Soil NH_4_^+^−N and NO_3_^−^−N contents were determined by auto-analyzer (Auto Analyzer 3, Bran Luebbe, Germany).

### 2.4 Statistical analysis

Plants were divided into three functional groups, including grasses, sedges, and forbs, to evaluate the impact of treatment on these functional groups.

The importance value (IV), species richness index *(R)*, and plant diversity index *(H)* were used as indicators of plant biodiversity and were calculated as follows:

IV:


$$IV=\frac{RH+RC}{2}$$

where *RC and RH* represent the mean values of relative cover and relative height.

Species richness index (*R*):


R=S

Plant diversity index (*H*):


$$H\;=-\sum_{i=1}^{S}(pi\ln pi)$$

where *S* represents the total number of species, *Pi* is the relative IV of the *i*th species.

To evaluate the differences in ecosystem function between litter removal and experimental warming treatments more accurately, we employed a Random Forest method to identify the ecological factors that contribute significantly to EMF. The results indicated that height, Cover, *R*, *H*, STN, SOM, NH_4_^+^–N, and pH are the primary predictors of ecosystem functions (**Supplementary Figure S5**). Thus, the aboveground ecosystem multifunctionality (AEMF) index was calculated by height, Cover, *R*, and *H*, while the belowground ecosystem multifunctionality (BEMF) index was calculated by STN, SOM, NH_4_^+^–N, and pH. Finally, these primary predictor factors were utilized to calculate the EMF index. The EMF index can be calculated in the following way (Maestre et al., 2012):


$$EMF=\frac{1}{N}\;\sum_{i=1}^{N}\;f(x_{i})$$

where N represents the number of functions and f(*x_i_*) is the standardization of the measured value obtained by:


$$f(xi)=(x_{i\;}-x_{mean})/std$$

where *x_i_*, *x_mean_* and *std* represents the measured value, mean value and standard deviation of all observations, respectively.

Structural equation modeling (SEM) was used to examine the direct and indirect effects of experimental warming treatment, litter removal treatment, plant productivity, plant diversity, and soil nutrients on EMF. Duncan method was used to analyze the significant differences in height, cover, IV, plant community characteristics, and soil physicochemical factors among different functional groups under various treatment conditions. Two factors analysis of variance was used to analyze the effects of experimental warming, litter removal, and their interaction on plant community characteristics and soil physicochemical factors. Pearson correlation is used to evaluate the relationship between individuals and multiple ecological functions. The SEM models were conducted using the AMOS 26 software, while other statistical analyses were executed using the SPSS 24.0 software. Graphics were accomplished using the Origin 2021 software.

## 3 Results

### 3.1 Plant community composition and structure under experimental warming, litter removal and experimental warming-litter removal

**Figure 2:**
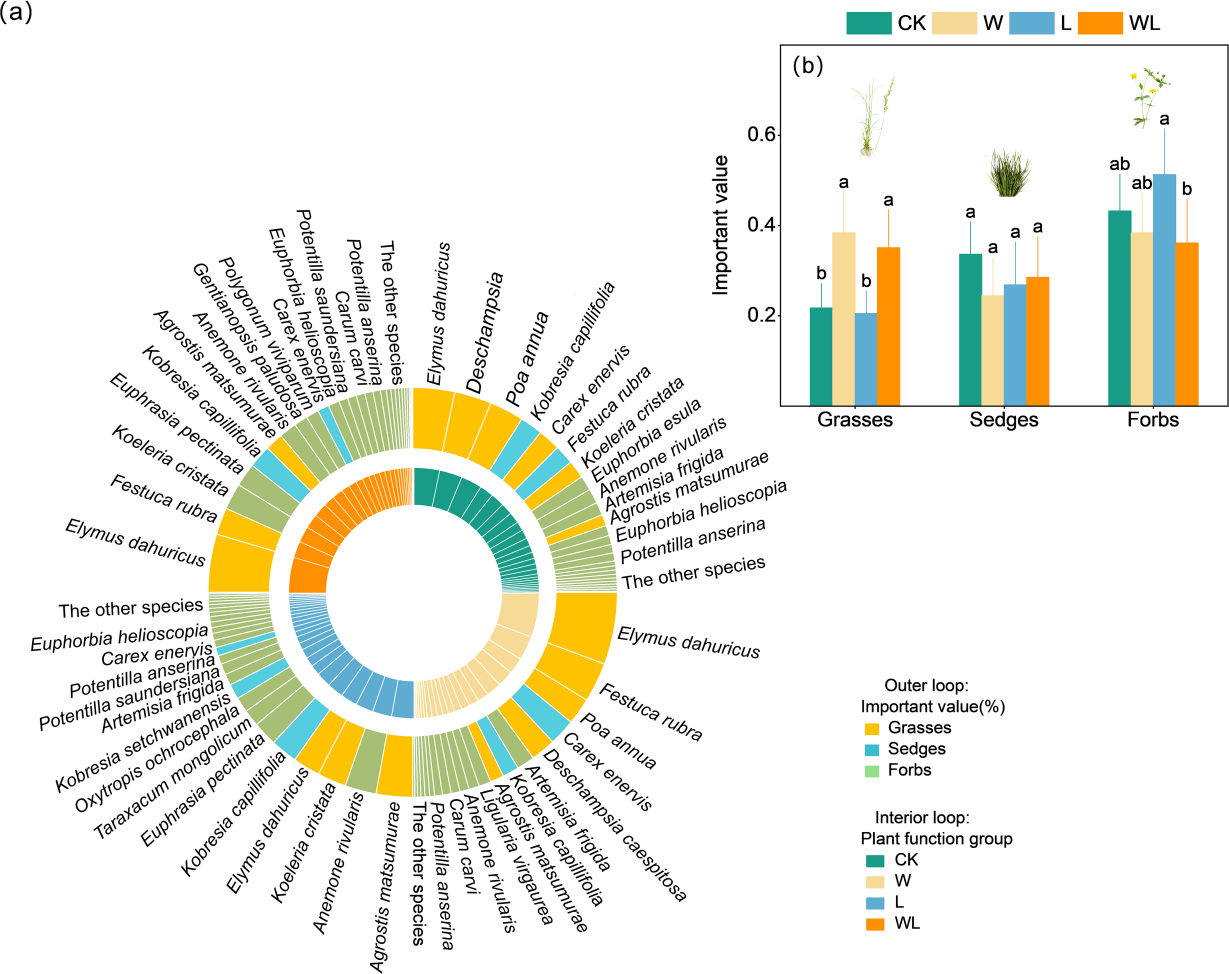
**(a)** The species and functional groups found in the present study. The colors of the internal loop represent the control (CK), experimental warming (W), litter removal (L) and experimental warming-litter removal (WL) treatments. In panel **(b)**, the colors of the external loop show the plant functional groups including sedges, grasses, and forbs. The areas of the loop segments are proportional to the relative importance value of each species. Different letters indicate significant differences among treatments.

The experimental warming treatment, experimental warming-litter removal treatment, and the control were dominated by *Elymus dahuricus*, with relative important values (IV) of 0.23, 0.18, and 0.13, respectively. However, litter removal treatment is primarily dominated by *Agrostis matsumurae*, with the IV of 0.12. In terms of functional groups, the grasses exhibited the highest dominance in control and experimental warming treatment, while the forbs exhibited the highest dominance in litter removal and experimental warming-litter removal treatment (**Figure 2a**). Overall, experimental warming, litter removal, and experimental warming-litter removal treatments all caused changes in each functional group. Specifically, compared with the control, experimental warming treatment resulted in a significant decrease in the height and cover of the sedges (*p* < 0.05) and a significant increase in the IV and cover of grasses (*p* < 0.05; **Supplementary Figure S4a**). The litter removal treatment led to a significant decrease in the height of the sedges (*p* < 0.05), while at the same time causing a significant increase in the cover of the forbs (*p* < 0.001; **Supplementary Figure S4b**). Experimental warming-litter removal treatment resulted in a significant decrease in the cover of sedges (*p* < 0.05), while simultaneously leading to a significant increase in the cover and IV of grasses (*p* < 0.05; **Supplementary Figure S4c**).

**Figure 3:**
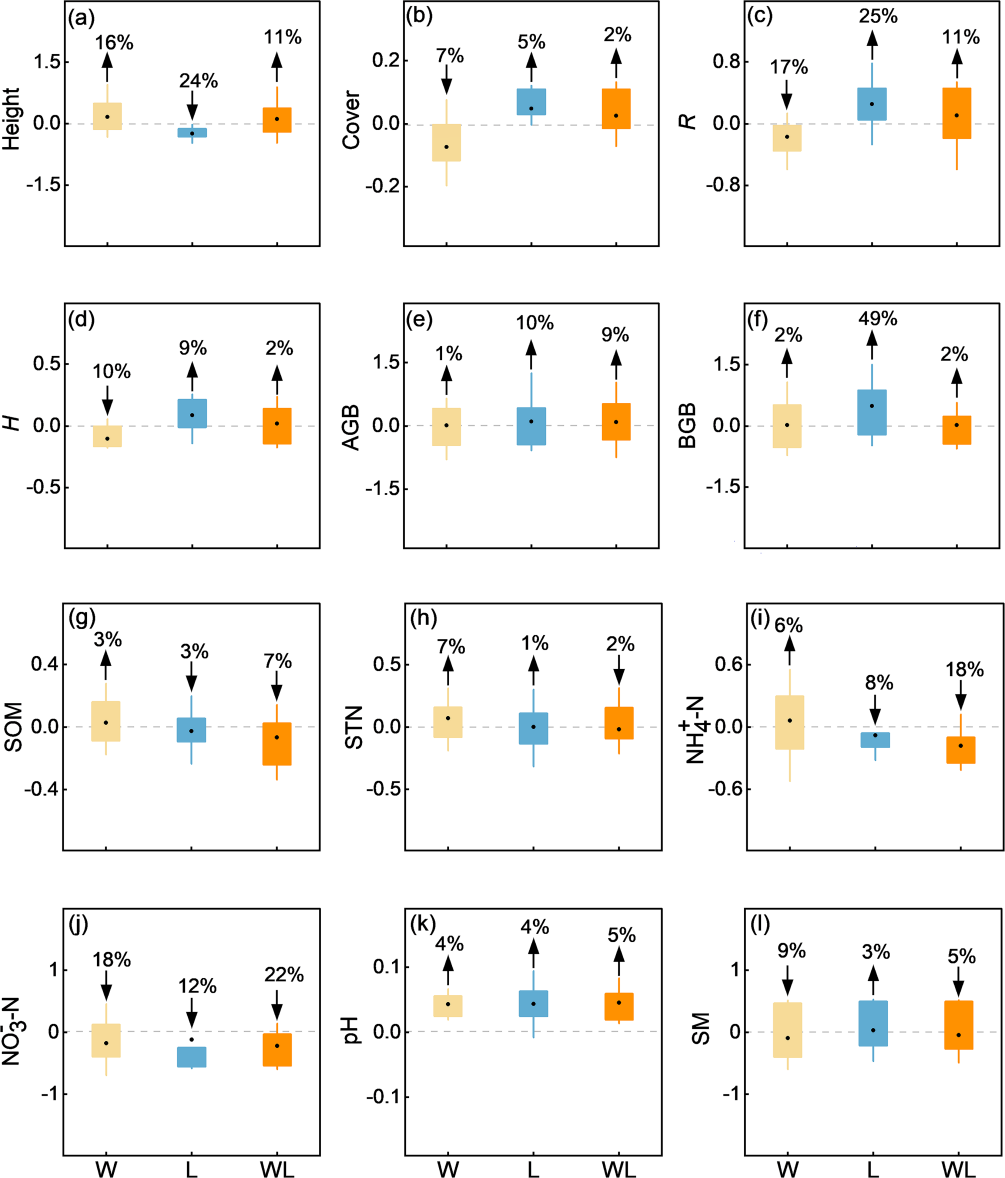
Differences in **(a)** community height (Height), **(b)** coverage (Cover), **(c)** species richness (*R*), **(d)** plant diversity (*H*), **(e)** aboveground biomass (AGB), **(f)** belowground biomass (BGB), **(g)** soil organic matter (SOM), **(h)** soil total nitrogen (STN), **(i)** soil ammonia nitrogen (NH_4_^+^–N), **(j)** soil nitrate nitrogen (NO_3_^–^N), **(k)** soil pH (pH), and **(l)** soil moisture content (SM) between experimental warming (W), litter removal (L), and experimental warming-litter removal (WL) treatments, compared to the control (CK).

**Figure 4:**
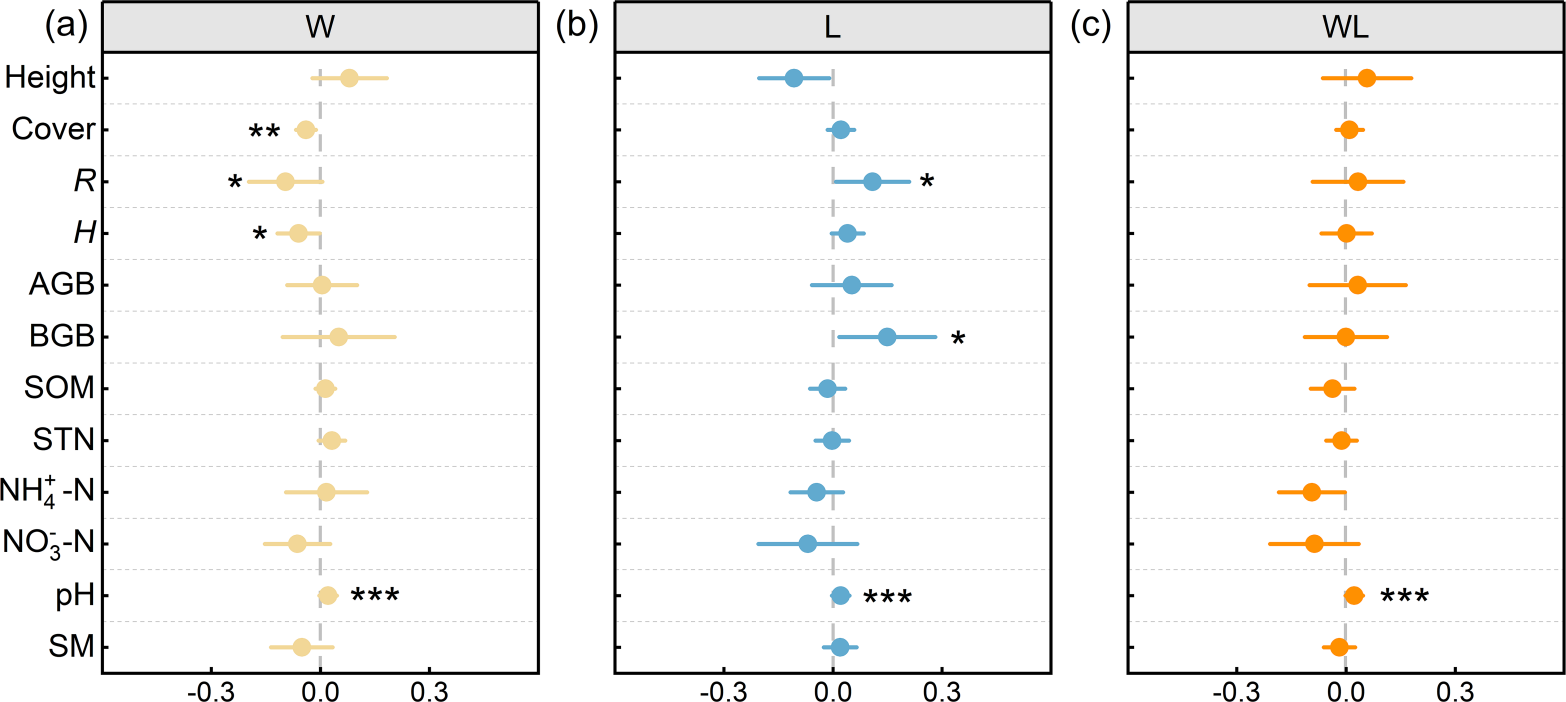
The effects of experimental warming (W), litter removal (L), and experimental warming-litter removal (WL) on plant communities and soil indicators. Asterisks indicate significant differences between each treatment (W, L, WL) and the control (CK). *, *p* < 0.05; **, *p* < 0.01; ***, *p* < 0.001.

The response of vegetation cover, height, AGB, BGB, *R*, and *H* to experimental warming treatment and litter removal treatment are inconsistent. Specifically, our results demonstrated a significant decrease in *R*, *H* (*p* < 0.05), and vegetation cover (*p*<0.01) under experimental warming treatment, with the average results showing that experimental warming treatment decreased the Cover, *R*, and *H* by an average of 7%, 17% and 10% (**Figures 3**, **4**). And the Height, AGB, BGB showed upward trend under experimental warming treatment (**Supplementary Figure S1**). The *R* and BGB significantly increased under litter removal treatment *(p* < 0.05; **Figure 4**). Litter removal treatment increased the *R* and BGB by an average of 25% and 49%, respectively (**Figure 3**). And the Height decreased, while Cover, *H*, and AGB showed an upward trend under litter removal treatment. The Height, Cover, *R*, *H*, AGB, and BGB all increased, but did not reach a significant level under experimental warming-litter removal treatment (**Supplementary Figure S1**).

### 3.2 Soil physical and chemical properties following experimental warming, litter removal and experimental warming-litter removal

The response of SOM, STN, NH_4_ *^+^*−N, NO_3_^−^−N, pH, and SM to experimental warming treatment and litter removal treatment is not consistent. Compared to the control, pH all significantly increased under experimental warming, litter removal, and experimental warming-litter removal treatments (*p* < 0.001; **Figure 4**). Results showed that the pH increased by 4% under experimental warming treatment and litter removal treatment. Experimental warming-litter removal treatment increased the pH by 5% (**Figure 3**). In addition, the SOM, STN, and NH_4_ *^+^*−N showed an upward trend under experimental warming treatment, meanwhile, the NO_3_^–^N and SM showed a downward trend. Under litter removal treatment, all the SOM, STN, NH_4_^+^–N, and NO_3_^–^N showed a downward trend, but the SM showed an upward trend. Under experimental warming-litter removal treatment, all the SOM, STN, NH_4_^+^−N, NO_3_^−^−N, and SM showed a downward trend (**Supplementary Figure S2**).

### 3.3 Changes in ecosystem functioning following experimental warming, litter removal and warming-litter removal

**Figure 5:**
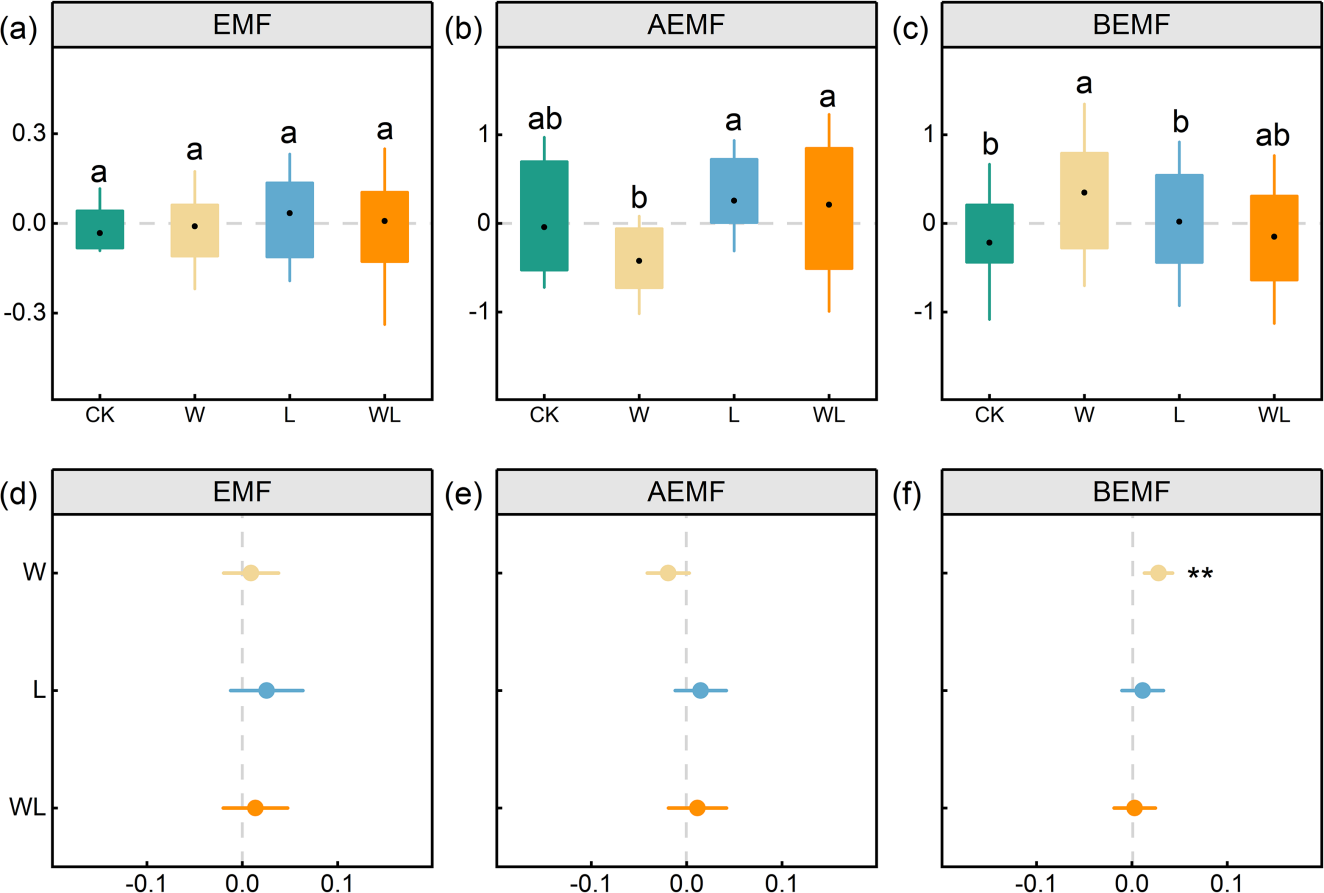
Changes of EMF (ecosystem multifunctionality), AEMF (aboveground ecosystem multifunctionality), BEMF (belowground ecosystem multifunctionality) in four different treatments plots **(a-c)**. Different letters indicate significant differences among treatments. The effects of experimental warming (W), litter removal (L), and experimental warming-litter removal (WL) on **(d)** EMF, **(e)** AEMF, **(f)** BEMF. Asterisks indicate significant differences between each treatment (W, L, WL) and the control (CK) *, *p* < 0.05; **, *p* < 0.01; ***, *p* < 0.001.

The EMF indexes of control, experimental warming treatment, litter removal treatment, and experimental warming-litter removal treatment were -0.26, -0.08, 0.27, and 0.06, respectively. Our results indicated that experimental warming, litter removal, and experimental warming-litter removal treatments had no significant effects on the EMF index. However, the aboveground ecosystem multifunctionality (AEMF) index and belowground ecosystem multifunctionality (BEMF) showed differential responses to experimental warming and litter removal treatments. The AEMF index showed a decreasing trend under experimental warming but showed an upward trend under both litter removal and experimental warming-litter removal treatments (**Figure 5b**). The BEMF index significantly increased under experimental warming (*p* < 0.01) but showed an upward trend under litter removal and experimental warming-litter removal treatments (**Figure 5c**). Under the experimental warming treatment, AEMF and BEMF exhibited opposite trends, which resulted in a balancing effect on EMF. Specifically, AEMF showed a decreasing trend, while BEMF increased significantly (*p* < 0.01), leading to an overall increase in EMF after balancing. In contrast, both AEMF and BEMF showed an increasing trend in litter removal and experimental warming-litter removal treatments, resulting in a corresponding increase in EMF.

### 3.4 Relationships of individual ecological functions with EMF, AEMF and BEMF

**Figure 6:**
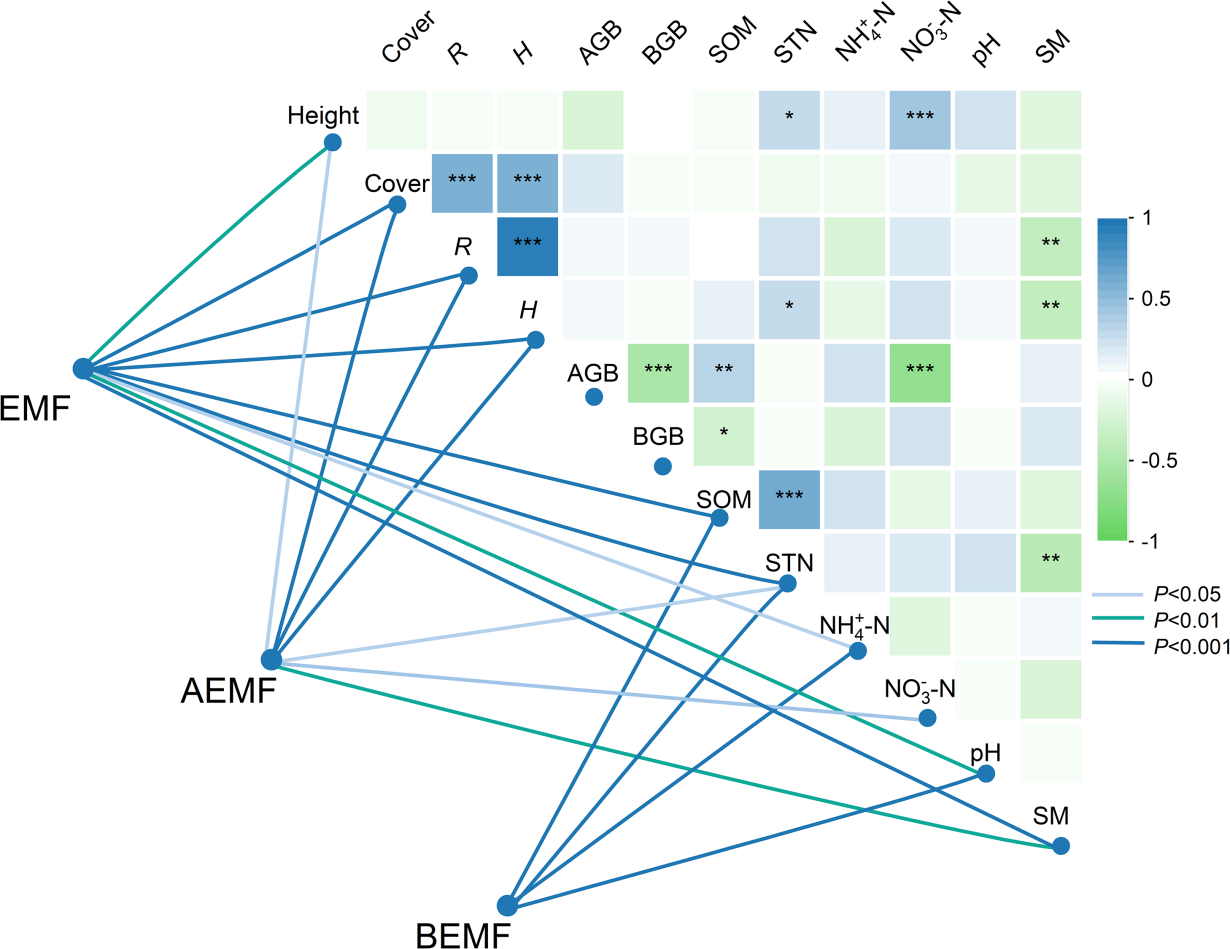
Correlation coefficients between the vegetation characteristics, soil properties, and the ecosystem multifunctionality (EMF) index. Cover, vegetation cover (%), Height, plant height (cm), *R*, species richness, *H*, plant diversity, AGB, aboveground biomass (g m^-2^), BGB, belowground biomass (g m^-2^), SOM, soil organic matter(SOM) (g kg^−1^), STN, soil total nitrogen (g kg^−1^), NH_4_^+^–N, soil ammonia nitrogen (mg kg^−1^), NO_3_^–^N, soil nitrate nitrogen (mg kg^−1^), pH, soil pH; SM, Soil moisture content (%). Asterisk was considered to be significant. *, *p* < 0.05; **, *p* < 0.01; ***, *p* < 0.001.

**Figure 7:**
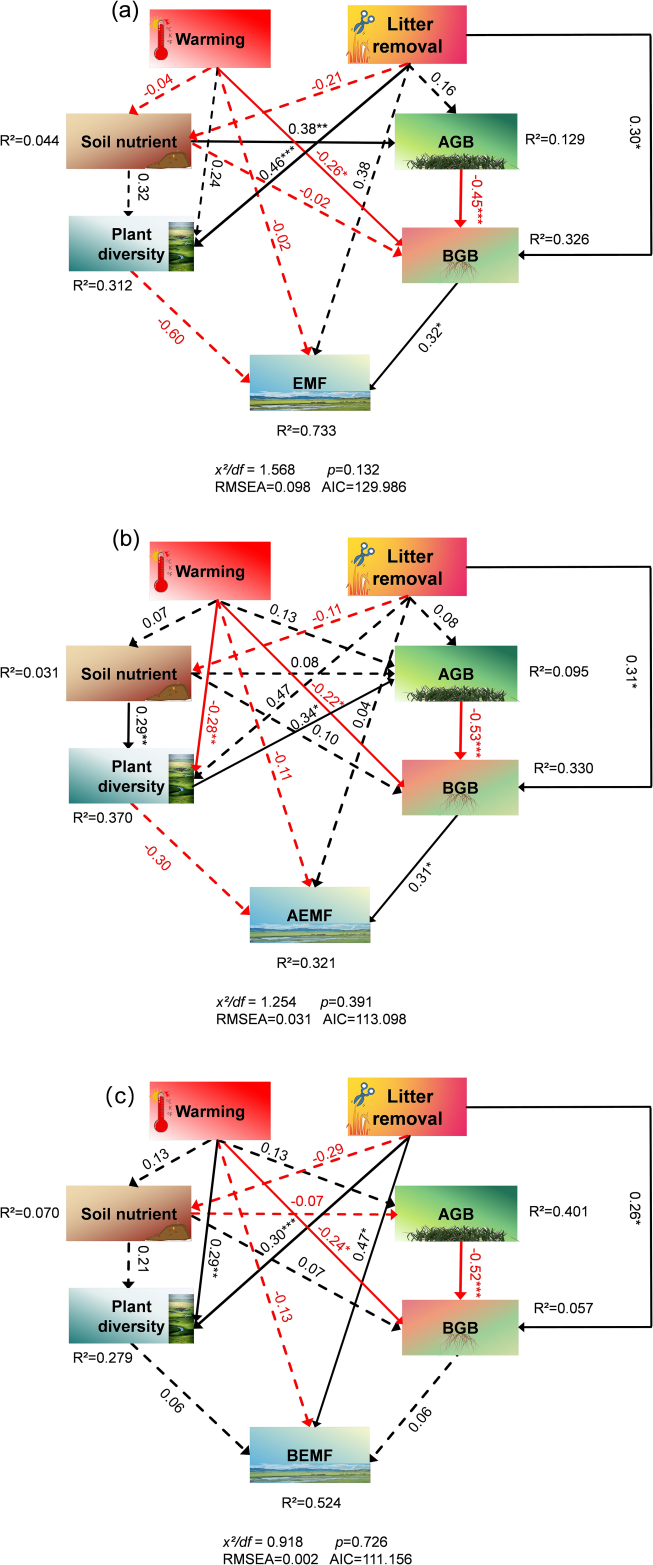
Effects of the experimental warming and litter removal on **(a)** ecosystem multifunctionality (EMF), **(b)** aboveground ecosystem multifunctionality (AEMF), **(c)** belowground ecosystem multifunctionality (BEMF) analyzed using structural equation model with path analysis based on observed data. Effects were calculated according to the standardized path coefficients. Black solid arrows represent positive effects. Red solid arrows represent negative effects. Significance is proportional to the thickness of the solid line. Dashed arrows indicate non-significant effects. Numbers next to arrows indicate standardized path coefficients. R^2^ represent the proportion of variance explained for each dependent variable in the model.

**Figure 8:**
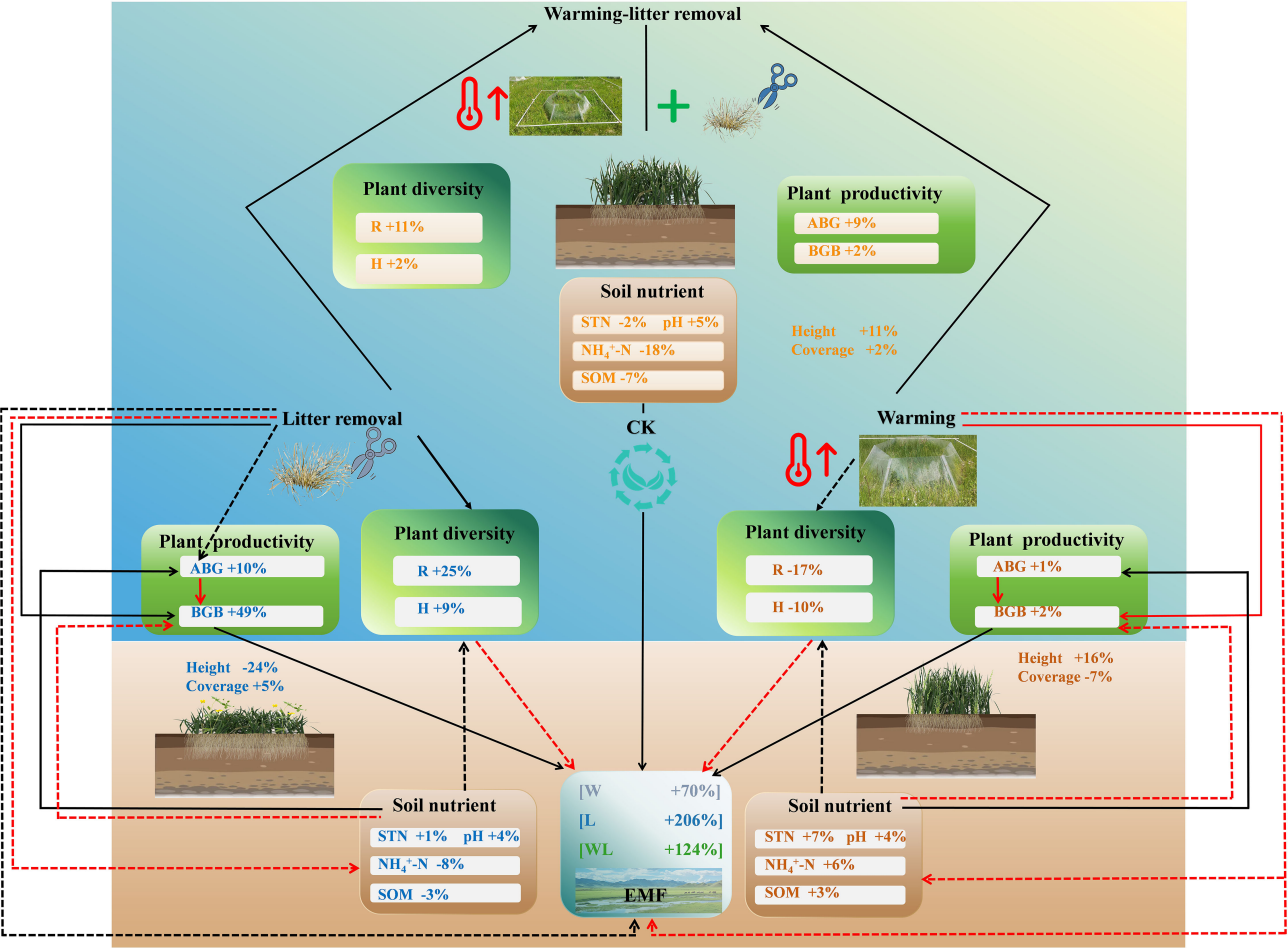
A conceptual framework diagram for understanding the potential effects of experimental warming and litter removal on the ecological functioning in semi-humid alpine grasslands. Black solid arrows represent positive effects. Red solid arrows represent negative effects. Dashed arrows indicate non-significant effects.

We analyzed the relationships among plant and soil functions, and the results showed that plant height was significantly correlated with STN and NO_3_^–^–N. The AGB was significantly correlated with SOM and NO_3_^–^–N. In addition, *H* was significantly correlated with STN and SM (**Figure 6**). We further analyzed the correlation between EMF, AEMF, and BEMF indexes with plant and soil functions. The results showed that EMF, AEMF, and BEMF indexes were significantly correlated with STN. The EMF index was significantly correlated with height, cover, *R*, *H*, SOM, STN, NH_4_^+^–N, pH, and SM, AEMF index was significantly correlated with height, cover, *R*, *H*, STN, NO_3_^–^N, and SM, BEMF index was significantly correlated with SOM, STN, NH_4_^+^–N, and pH (**Figure 6**). Path analysis of the structural equation model showed that experimental warming and litter removal treatments influence the EMF and AEMF indexes mainly directly by affecting BGB. In the total impact on EMF and BEMF index, the contribution of soil nutrients is much higher than other indicators, while in the AEMF index, the contributions of AGB and BGB are higher than the other functions (**Figures 7**, **8**).

## 4 Discussion

### 4.1 The impact of experimental warming on ecosystem structure and function

Temperature is a pivotal factor influencing plant growth and development. In cold and high-altitude regions, climate warming is more likely to impact the structure and traits of plant communities (Quan et al., 2024). The main reason for this result is that at high altitudes, certain highly temperature-sensitive plant species are more vulnerable to climate change, leading to species decline or extinction, which further leads to changes in community composition and structure (Ma et al., 2017). Our findings indicate that under experimental warming treatment, both the cover and importance value (IV) of grasses significantly increased (*p* < 0.05), while the cover and height of sedges significantly decreased (*p* < 0.05; **Supplementary Figures S3**, **S4**). The reason for this result is the difference in resistance between sedges and grasses. Warming could increase the abundance of grasses at the expense of the biomass of sedge and other non-grasses (Liu et al., 2018). On the one hand, the response of sedges to climate warming is primarily due to moisture limitation, the decrease in moisture levels caused by climate warming has been shown to have a detrimental effect on the growth of sedges (Yuan et al., 2021); on the other hand, the low-temperature limitation of alpine meadows is broken by warming, and the increase in vegetation height and AGB enhances the ability to compete for light, with sedges and some low forbs being excluded from competition by grasses (Hu et al., 2021). For a specific functional group type, warming may have opposite effects on the relative abundance between species (Ganjurjav et al., 2016). This finding indicates that functional groups may not adequately represent changes at the species level, thus emphasizing the necessity for further research in this area. Additionally, changes in vegetation functional groups can also explain the response of cover to warming (Niu et al., 2025), which means that warming decreases plant diversity and further affects cover.

Research on alpine meadows has revealed that, under 20 years of experimental warming treatment, the effect of temperature on soil pH increase showed significant differences (Alatalo et al., 2017). Our results indicate that experimental warming significantly increased soil pH (*p* < 0.001; **Figure 4a**). Changes in microbial activity as a result of warming, where microbial decomposition of organic matter releases alkaline compounds, can be used to explain the increase in pH (Han et al., 2022). Meanwhile, under warming, the soil moisture content decreases (**Supplementary Figure S2**). Warming-induced declines in soil moisture are usually accompanied by changes in nitrification intensity, which affects the conversion of NH_4_^+^ and NO_3_^-^, while increased nitrogen leaching and gaseous nitrogen losses accelerate the depletion of soil nitrogen pools (Fang et al., 2023). The observed decreases in NO_3_^–^–N and SM in this study indicate a response to warming. However, the absence of significant changes may be attributed to the relatively short duration of the experimental warming treatment. Therefore, future long-term monitoring is essential for a deeper understanding of nutrient cycling responses to climate warming.

### 4.2 The impact of litter removal on ecosystem structure and function

The results of this study indicate that under litter removal, the height and IV of grasses have exhibited a declining trend, while the cover of forbs has significantly increased (*p* < 0.001), and IV of forbs has shown an upward trend (**Supplementary Figure S4**). The following factors are considered to be the underlying causes of the observed changes in functional groups. Firstly, with litter removal, sedges and low-growing forbs received sufficient light, which significantly enhanced their dominance in the grass layer (Wu et al., 2004). Meanwhile, litter removal can affect the nutritional utilization strategies of plants, indirectly affecting their competitiveness and biomass of plants. It is evident that litter removal dramatically reduces the dominant position of dominant species in grasslands (Li et al., 2020). In contrast, the dominance of secondary and associated species increases, affecting grassland productivity and species diversity and further affecting the EMF.

In the analysis of soil properties, the decomposition process of litter releases organic acids. In this experiment, litter removal resulted in a decrease in organic acid content, leading to a significant increase in pH (*p* < 0.05; **Figure 4a**). In addition, litter can be leached and decomposed to provide soluble nutrients to the soil directly and nitrogen content is the main influencing factor in the process of litter decomposition (Błońska et al., 2021); in this study, there was no significant change in soil total nitrogen content and soil organic matter under litter removal, which is consistent with the previous research results (Lajtha et al., 2014). This can be attributed to the relatively slow mineralization and nutrient accumulation process of soil organic matter, so the short-term effect of litter removal on soil total nitrogen content may not yet be apparent. Regarding the analysis of soil available nitrogen, previous studies have found that litter removal will reduce the soil nitrogen pool and increase NO_3_^−^ leaching, indicating that litter removal decreases the storage and availability of nitrogen in the soil (Zhang et al., 2023b); our research findings also demonstrate that litter removal results in a decrease in NH_4_^+^–N and NO_3_^–^–N, although these changes did not achieve statistical significance, this may be because the duration of litter removal was relatively short and has not yet had a significant impact on soil NH_4_^+^–N and NO_3_^–^–N.

### 4.3 Controls of the multiple ecosystem functions under experimental warming and litter removal

In fact, ecosystem function can be influenced by many factors, such as climate conditions, biodiversity levels, and even experimental design. Most previous studies have shown a general positive correlation between plant diversity and ecosystem functions, indicating that plant diversity has a limiting effect on ecosystem functions, as communities with high species diversity can maintain more functions at high levels (Xu et al., 2021). Research has demonstrated that litter indirectly drives changes in EMF by mediating plant cover and species richness in alpine meadows (Ma et al., 2021). Moreover, plant litter affects changes in soil physico-chemical properties in alpine meadows, thereby impacting ecosystem function (Tian et al., 2021). The findings of our study demonstrated that the response of AEMF to litter removal was predominantly contingent on alterations in plant diversity and that diversity exhibited a significant correlation with ecosystem function (**Figure 6**). The positive responses of ecosystem functions to litter removal can be explained by the litter removal-induced increases in primary productivity and species diversity and decreases in soil nutrient. The improvement in productivity and diversity offsets the adverse effects of other ecosystem functions, ultimately leading to an upward trend in ecosystem functions because of litter removal.

Studies have shown that experimental warming can influence EMF by affecting the plant diversity and productivity of alpine grasslands (Zhao et al., 2024). A plethora of research has demonstrated a correlation between plant diversity and vegetation productivity in alpine grasslands and ecosystem function (Jing et al., 2015). Plant diversity is identified as the key factor mediating the effects of human activities on EMF (Zhang et al., 2021), and may decrease with increasing temperature (Ma et al., 2022), further impacting the ecosystem functions. Specifically, due to some species being more sensitive to warming than others, warming can reduce the species richness (Gruner et al., 2017) and alter species composition (Perkins et al., 2010), affecting both individual ecosystem functions and overall multifunctionality (Perkins et al., 2010, 2015). Our study has demonstrated that experimental warming would primarily affect the AEMF through alterations to plant diversity and vegetation productivity (**Figure 7**). In addition, the correlation between productivity and diversity may also change under climate change. Warming has altered the positive correlation between AGB and species diversity in alpine grasslands under natural conditions, thereby weakening the dependence of AGB on species diversity (Ma et al., 2022). Warming reduces the correlation between species diversity and vegetation productivity, suggesting that environmental factors significantly impact the relationship between the two. In conclusion, experimental warming decreased diversity and cover (**Figure 4a**), which further affected AEMF by affecting productivity (**Figure 7**). Research demonstrates that soil pH significantly correlates with EMF in alpine meadows and that it indirectly influences EMF through structural, compositional, and functional modulation of soil communities (Shi et al., 2024; Hu et al., 2024). In addition, nitrogen availability has been shown to mediate EMF through coordinated regulation of species richness and functional diversity (Pichon et al., 2024). Specifically in alpine meadows, experimental analyses reveal that nitrogen-induced variations in multifunctionality are fundamentally governed by plant diversity-mediated ecological mechanisms (Liu et al., 2021). The decrease in moisture caused by warming led to the migration of nitrogen pools (Fang et al., 2023), while the increase in pH, SOM, and NH_4_^+^–N affected BEMF. The positive effects of soil nutrients offset the negative impacts on other ecosystem functions, ultimately achieving a balance in ecosystem functions. In the interaction between experimental warming and litter removal, litter removal diminished the limiting effects of warming, enhanced the *R* and *H*, and consequently influenced AEMF by affecting AGB and BGB. Ultimately, this led to litter removal, mitigating the decline in aboveground ecosystem multifunctioning caused by warming. In addition, it has been shown that litter is closely related to the formation of SOC (Angst et al., 2021), our results confirm that litter removal leads to a downward trend in SOC (**Figure 3**). However, the significant increase in pH offset the decreasing trend of other soil nutrients, and BEMF still showed an increasing trend under litter removal treatment. Therefore, the interaction between experimental warming and litter removal promoted upward trends in AEMF and BEMF by mediating plant diversity, productivity, and soil nutrients, thereby further enhancing EMF.

### 4.4 Limitations and future research

The present research has certain limitations that should be acknowledged. Our study plots were established within fenced areas, and grazing exclusion may affect changes in ecosystem functions. Therefore, grazing exclusion and the two experimental treatments (experimental warming and litter removal) may exert interactive effects that could confound the interpretation of experimental outcomes. In addition, litter removal can serve as an experimental treatment to simulate grazing, and the results of this experiment explain the effects of grazing on EMF to a certain extent. However, the actual effects depend on factors such as environmental conditions, soil type, and plant species. Our future studies should focus on the individual and interactive effects of experimental warming, different grazing intensities, and grazing exclusion on alpine grassland ecosystems.

Another important point to note is that the duration of the experimental warming and litter removal experiments in this study was relatively short, which may limit our ability to assess fully the effects of these two factors on ecosystem functioning. Therefore, future research should focus on simulating the long-term effects of experimental warming and litter removal on individual and multiple ecosystem functions. This will help elucidate the specific mechanisms underlying ecosystem responses to climate change and provide more NbS, such as litter management measures to enhance ecosystem functionality.

## 5 Conclusions

Our findings provided novel experimental evidence that litter removal could mitigate the negative effects of warming on ecosystem functions in semi-humid alpine grasslands. This response is mainly driven by the direct and indirect effects of warming and litter removal on plant diversity, productivity, and soil nutrients. However, warming affected the aboveground and belowground ecosystem functions of alpine grasslands differently, with the AEMF index decreasing while the BEMF index increased under experimental warming. Consequently, the balancing effect of litter removal on ecological functions varies, which is primarily reflected in the AEMF index in alpine grasslands.

Our findings highlight the importance of plant litter removal in maintaining ecological functions by altering plant diversity and productivity in semi-humid alpine grasslands under climate change. We further emphasize the need to implement appropriate grassland management measures to regulate plant litter accumulation and apply suitable litter management practices along with other NbS to enhance ecosystem functionality. These practices include controlling grazing intensity to maintain the balance of plant communities and prevent excessive litter build-up, as well as applying scientific fertilization and improving soil quality to accelerate litter decomposition. By adopting these measures, we can enhance ecosystem biodiversity and the multifunctionality of alpine grasslands in semi-humid areas of the QTP, thereby further strengthening the capacity of grassland ecosystems to cope with climate change.

## Data Availability

The raw data supporting the conclusions of this article will be made available by the authors, without undue reservation.

## Supplementary material

Supplementary Figure 1 Differences in **(a)** plant height (Height), **(b)** vegetation coverage (Cover), **(c)** species richness (*R*), **(d)** plant diversity (*H*), **(e)** aboveground biomass (AGB), and **(f)** belowground biomass (BGB) among the control (CK), experimental warming (W), litter removal (L), and experimental warming-litter removal (WL) treatments. Different letters indicate significant differences among four treatments.

Supplementary Figure 2 Differences in **(a)** soil organic matter (SOM), **(b)** soil total nitrogen (STN), **(c)** soil ammonia nitrogen (NH_4_^+^–N), **(d)** soil nitrate nitrogen (NO_3_^–^N), **(e)** soil pH and **(f)** soil moisture content (SM) among the control (CK), experimental warming (W), litter removal (L) and experimental warming-litter removal (WL) treatments. Different letters indicate significant differences among four treatments.

Supplementary Figure 3 Differences in plant height (Height), vegetation coverage (Cover), Important value of three different vegetation functional groups including sedges, grasses and forbs among the control (CK), experimental warming (W), litter removal (L) and experimental warming-litter removal (WL) treatments. Different letters indicate significant differences among four treatments.

Supplementary Figure 4 The effects of experimental warming (W), litter removal (L), and experimental warming-litter removal (WL) on plant height (Height), vegetation coverage (Cover), and important value (IV) of three vegetation functional groups, including sedges, grasses, and forbs. Asterisks indicate significant differences between each treatment (W, L, WL) and the control (CK). *, *p* < 0.05; **, *p* < 0.01; ***, *p* < 0.001.

Supplementary Figure 5 A total of 8 factors with significant influence on EMF were screened out by a random forest model, including: plant diversity (*H*), soil total nitrogen (STN), soil organic matter (SOM), species richness (*R*), vegetation coverage (Cover), soil ammonia nitrogen (NH_4_^+^–N), plant height (Height), and soil pH (pH).
