# Supplementary figures and images for: Effects of simulated warming and litter removal on structure and function of semi-humid alpine grassland in the Qinghai-Tibet Plateau
Source: Front Plant Sci. 2025 May 15;16:1567414. doi: 10.3389/fpls.2025.1567414 (PMC12119568; doi:10.3389/fpls.2025.1567414)

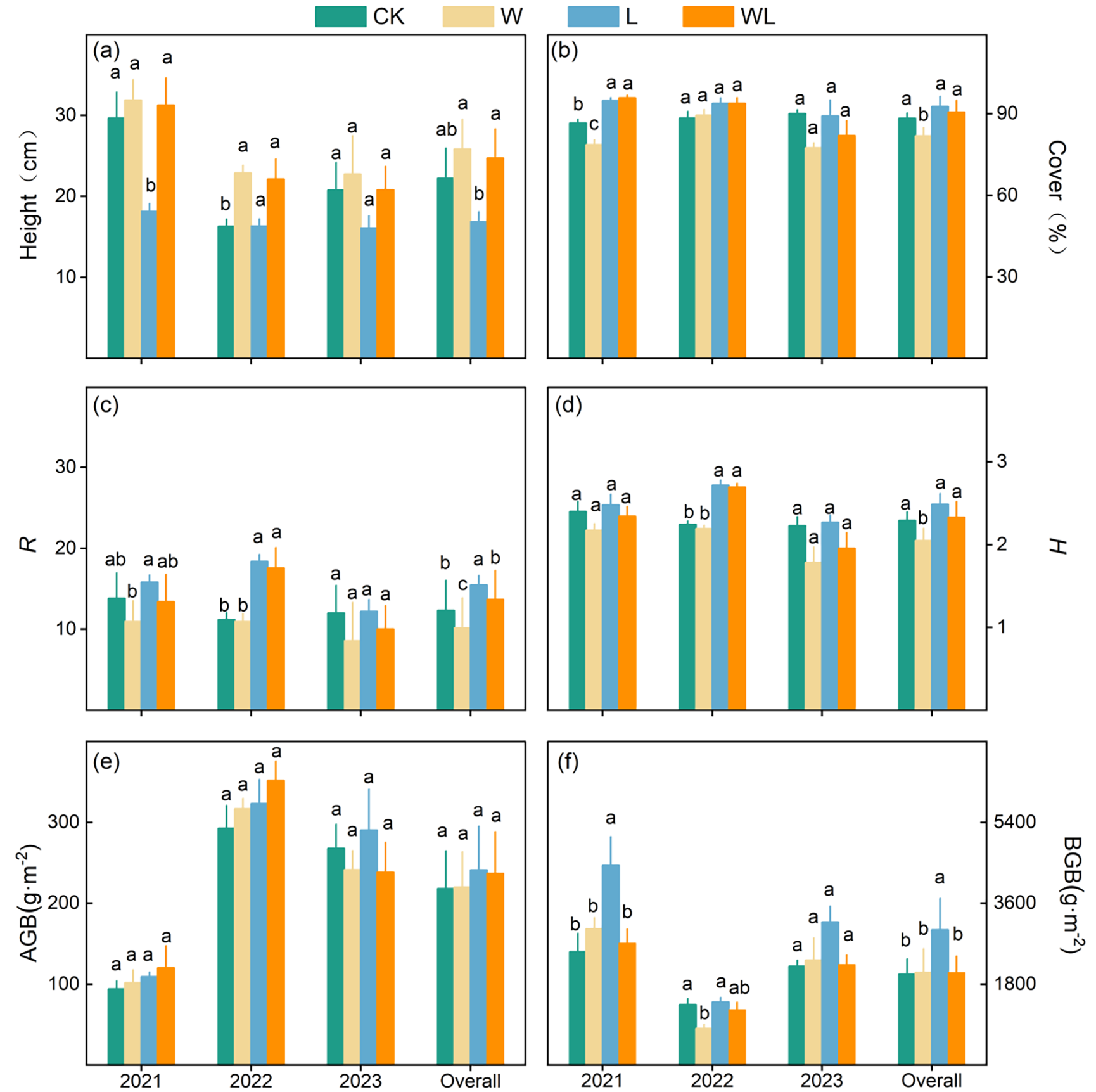

Supplement: Supplementary Figure 1 — Differences in (a) plant height (Height), (b) vegetation coverage (Cover), (c) species richness (R), (d) plant diversity (H), (e) aboveground biomass (AGB), and (f) belowground biomass (BGB) among the control (CK), experimental warming (W), litter removal (L), and experimental warming-litter removal (WL) treatments. Different letters indicate significant differences among four treatments. [file Image1.jpeg]

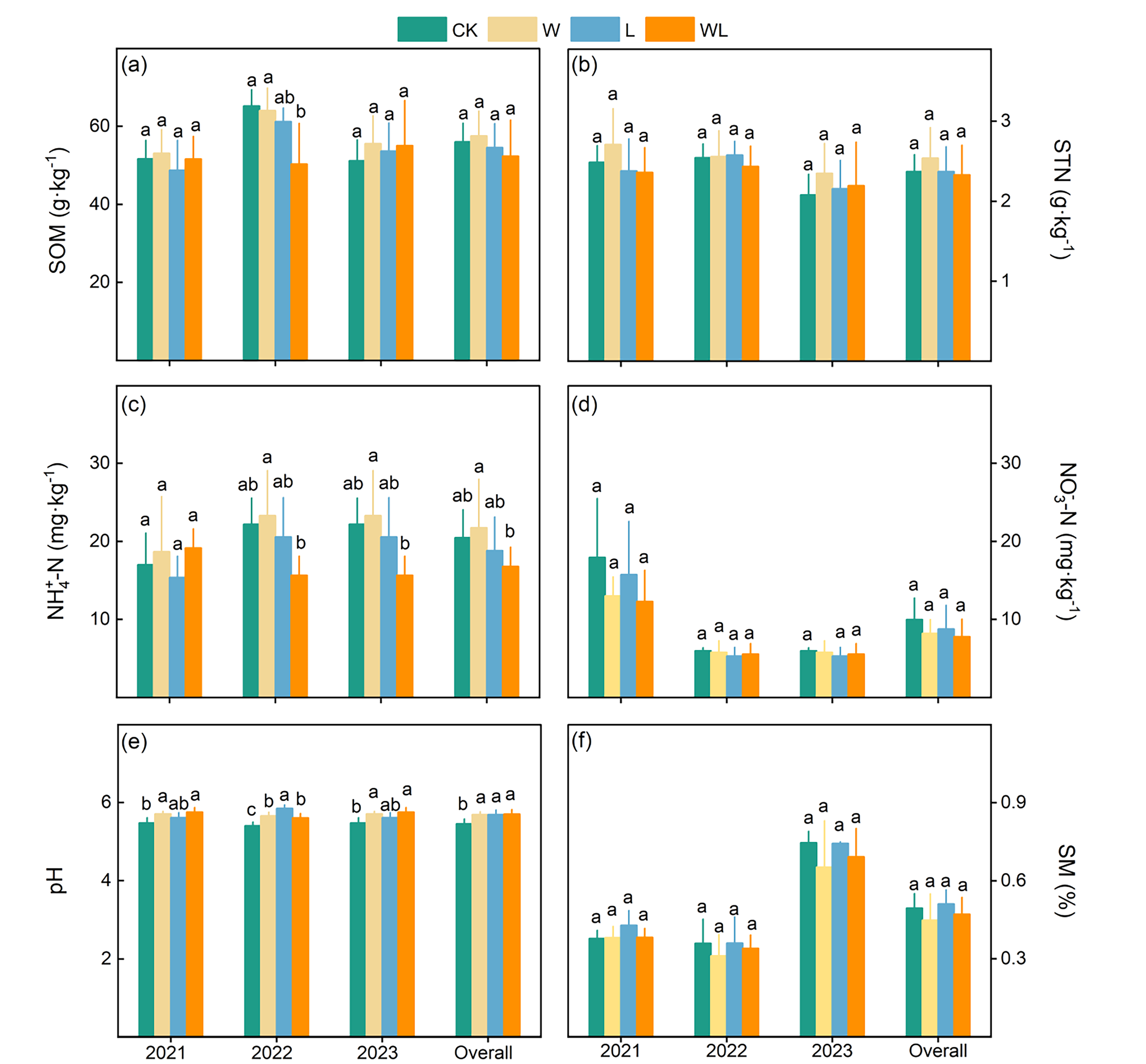

Supplement: Supplementary Figure 2 — Differences in (a) soil organic matter (SOM), (b) soil total nitrogen (STN), (c) soil ammonia nitrogen (NH4 +–N), (d) soil nitrate nitrogen (NO3 –N), (e) soil pH and (f) soil moisture content (SM) among the control (CK), experimental warming (W), litter removal (L) and experimental warming-litter removal (WL) treatments. Different letters indicate significant differences among four treatments. [file Image2.jpeg]

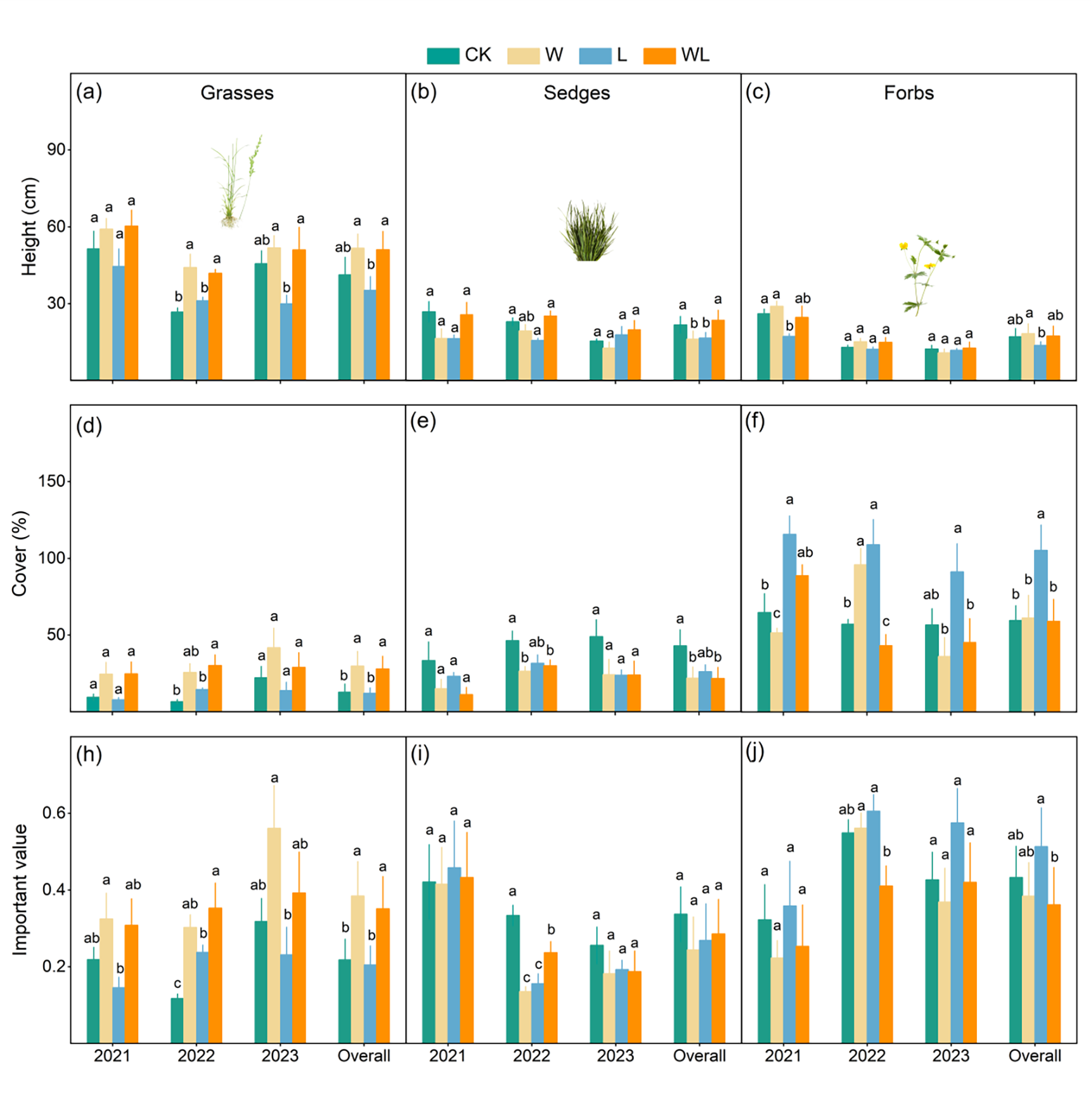

Supplement: Supplementary Figure 3 — Differences in plant height (Height), vegetation coverage (Cover), Important value of three different vegetation functional groups including sedges, grasses and forbs among the control (CK), experimental warming (W), litter removal (L) and experimental warming-litter removal (WL) treatments. Different letters indicate significant differences among four treatments. [file Image3.jpeg]

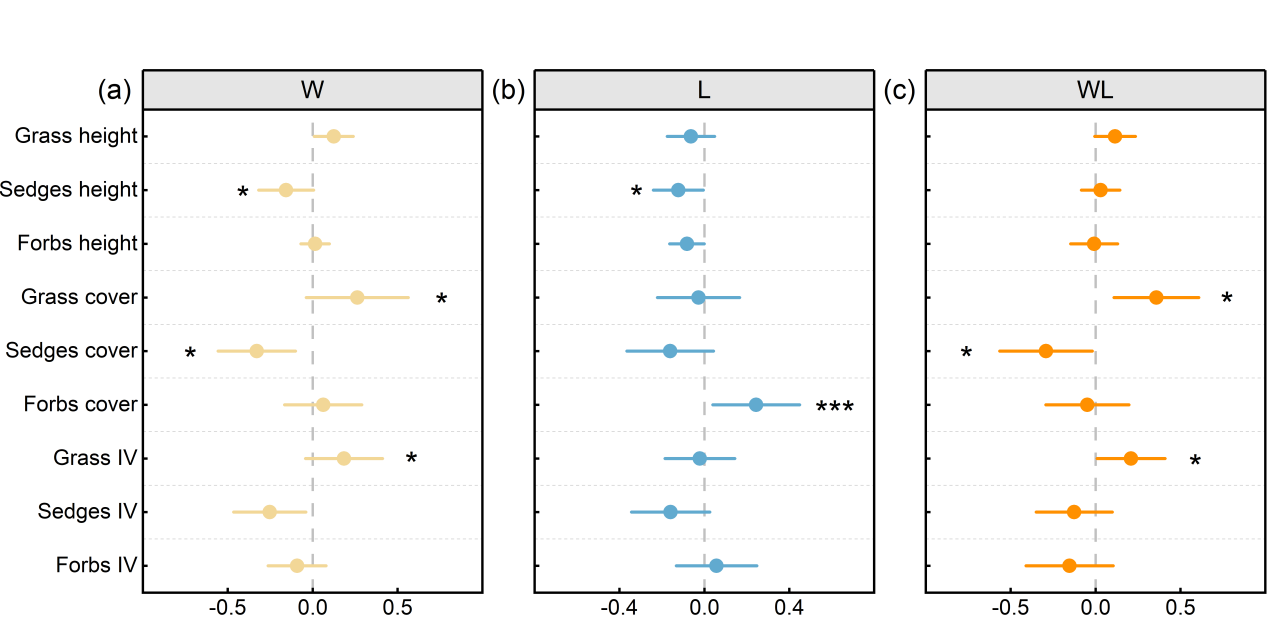

Supplement: Supplementary Figure 4 — The effects of experimental warming (W), litter removal (L), and experimental warming-litter removal (WL) on plant height (Height), vegetation coverage (Cover), and important value (IV) of three vegetation functional groups, including sedges, grasses, and forbs. Asterisks indicate significant differences between each treatment (W, L, WL) and the control (CK). *, p < 0.05; **, p < 0.01; ***, p < 0.001. [file Image4.jpeg]

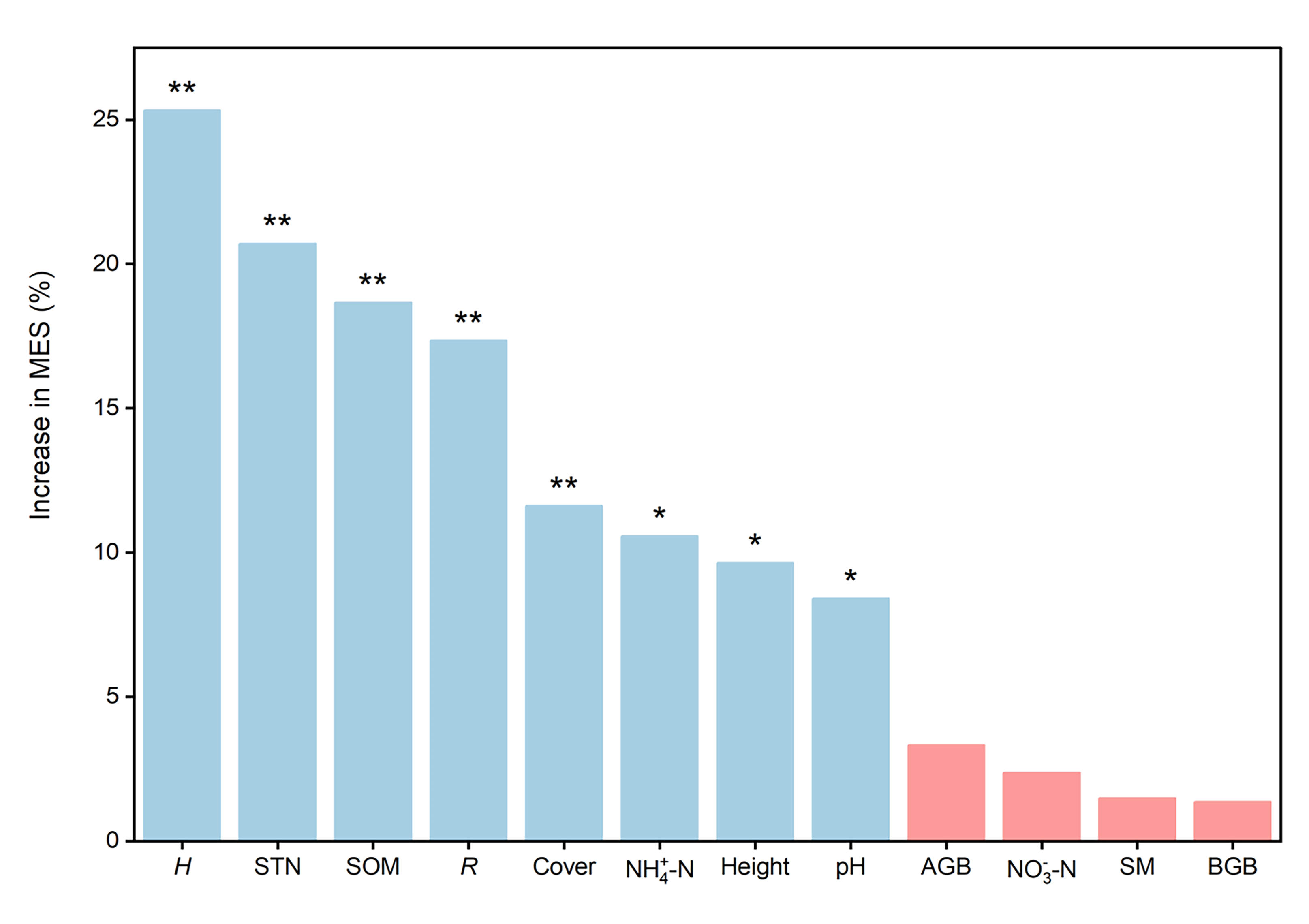

Supplement: Supplementary Figure 5 — A total of 8 factors with significant influence on EMF were screened out by a random forest model, including: plant diversity (H), soil total nitrogen (STN), soil organic matter (SOM), species richness (R), vegetation coverage (Cover), soil ammonia nitrogen (NH4 +–N), plant height (Height), and soil pH (pH). [file Image5.jpeg]
